# Predicting grip strength-related frailty in middle-aged and older Chinese adults using interpretable machine learning models: a prospective cohort study

**DOI:** 10.3389/fpubh.2024.1489848

**Published:** 2024-12-17

**Authors:** Lisheng Yu, Shunshun Cao, Botian Song, Yangyang Hu

**Affiliations:** ^1^Neurosurgery, The Second Affiliated Hospital of Wenzhou Medical University, Wenzhou, China; ^2^Wenzhou Municipal Key Laboratory of Neurodevelopmental Pathology and Physiology, Wenzhou Medical University, Wenzhou, China; ^3^Pediatric Endocrinology, Genetics and Metabolism, The Second Affiliated Hospital of Wenzhou Medical University, Wenzhou, China; ^4^Reproductive Medicine Center, Obstetrics and Gynecology, The Second Affiliated Hospital of Wenzhou Medical University, Wenzhou, China

**Keywords:** frailty, grip strength, machine learning, SHapley Additive exPlanation, CHARLS

## Abstract

**Introduction:**

Frailty is an emerging global health burden, and there is no consensus on the precise prediction of frailty. We aimed to explore the association between grip strength and frailty and interpret the optimal machine learning (ML) model using the SHapley Additive exPlanation (SHAP) to predict the risk of frailty.

**Methods:**

Data for the study were extracted from the China Health and Retirement Longitudinal Study (CHARLS) database. Socio-demographic, medical history, anthropometric, psychological, and sleep parameters were analyzed in this study. We used the least absolute shrinkage and selection operator (LASSO) regression to filter the model for the best predictor variables and constructed six ML models for predicting frailty. The feature performance of six ML models was compared based on the area under the receiver operating characteristic curve (AUROC) and the light gradient boosting machine (LightGBM) model was selected as the best predictive frailty model. We used SHAP to interpret the LightGBM model and to reveal the decision-making process by which the model predicts frailty.

**Results:**

A total of 10,834 eligible participants were included in the study. Using the lowest quartile of grip strength as a reference, grip strength was negatively associated with the risk of frailty when grip strength was >29.00 kg for males or >19.00 kg for females (*p* < 0.001). The LightGBM model predicted frailty with optimal performance with an AUROC of 0.768 (95% CI 0.741 ~ 0.795). The SHAP summary plot showed that all features predicted frailty in order of importance, with cognitive function being considered the most important predictive feature. The poorer the cognitive function, nighttime sleep duration, body mass index (BMI), and grip strength, the higher the risk of frailty in middle-aged and older adults. The SHAP individual force plot clearly shows that the LightGBM model predicts frailty in the individual decision-making process.

**Conclusion:**

The grip strength-related LightGBM prediction model based on SHAP has high accuracy and robustness in predicting the risk of frailty. Increasing grip strength, cognitive function, nighttime sleep duration, and BMI reduce the risk of frailty and may provide strategies for individualized management of frailty.

## 1 Introduction

Frailty is a complex, multidimensional biopsychosocial syndrome associated with aging, encompassing the activity, physiological, cognitive, social, and psychological domains, and characterized by a decline in the physiological capacity of multiple organ systems leading to increased sensitivity to stressors (1). Epidemiologic surveys of people aged 50 years and older in 15 countries showed an average prevalence of frailty of 17.4% (ranging from 3.9 to 51.4%), with a higher prevalence of frailty among older persons in upper-middle-income countries than in high-income countries (2). A prospective study of people over 85 years of age found that frail patients had more than twice the risk of death as non-frail patients at 7 years, and that frailty led to a 54 to 101% increase in healthcare expenditures (3). Therefore, early recognition and treatment of frailty have important public health and clinical implications.

Currently, several well-established methods are available to assess frailty, each offering distinct strengths and limitations. The frailty phenotype model, introduced by Fried LP et al., characterizes frailty based on five physical criteria: unintentional weight loss, exhaustion, low physical activity, slow walking speed, and weak grip strength (4). This model is widely adopted due to its simplicity and emphasis on physical attributes. In contrast, the cumulative deficit model, developed by Rockwood et al., evaluates frailty by tallying health deficits across multiple domains—including physical, cognitive, and social factors—to calculate a frailty index (5). Additionally, tools such as the Tilburg Frailty Indicator (TFI) and the Edmonton Frail Scale (EFS) provide multidimensional assessments that encompass psychological and social domains (6).

Among these methods, grip strength has been extensively validated as a simple, objective, and reliable biomarker for assessing physical frailty. Its use is particularly advantageous in large-scale cohort studies due to its ease of measurement and established association with overall health status (7). Studies have shown grip strength to be generally consistent with the assessment of overall body strength, upper extremity function, fractures, bone density, falls, aging, malnutrition, cognitive dysfunction, depression, sleep disorders, diabetes mellitus, and co-morbidities, and to be associated with all-cause mortality and disease-specific mortality (8). A Mendelian randomization study showed that grip strength was positively and causally related to lumbar spine bone density at the best incidence of osteoporotic fracture, but not to heel forearm or femoral neck bone density (9). A review of the relationship between grip strength and diabetes mellitus by Hamasaki H et al. suggests that grip strength is a reliable indicator for identifying the risk of diabetes mellitus, cardiovascular disease, and mortality, as well as for assessing skeletal muscle mass and strength (10). In addition, Hadzibegovic S et al. concluded that grip strength can also be used to assess the strength and functional status of cancer patients, especially those with cancer cachexia, and is a widely used functional test (11).

Machine learning (ML) represents a powerful tool in predictive disease modeling, offering significant advantages over traditional statistical methods, especially in handling complex, high-dimensional datasets (12). There are many different algorithms for ML, each with different characteristics for analyzing data. Various ML methods have been widely applied in health research, including Support Vector Machine (SVM) known for its classification precision in high-dimensional spaces, Random Forest (RF) for its robustness in variable selection, and Gradient Boosting methods like Light Gradient Boosting Machine (LightGBM) and Extreme Gradient Boosting (XGBoost) for their superior performance in imbalanced datasets. Interpretable ML overcomes the shortcomings of the black-box nature of ML, which allows users to better understand the underlying logic of the predictive model, increases the credibility of the predictive model, and enables the timely detection of possible biases in the model, as well as the diagnosis and repair of the model (13). To the best of our knowledge, no studies have explored grip strength as a predictor of frailty in middle-aged and older Chinese using interpretable ML methods and explored the relationship between grip strength and frailty. To fill this gap, we hypothesized that grip strength could be used as an independent predictor of frailty in middle-aged and older Chinese adults. Based on the data from the China Health and Retirement Longitudinal Study (CHARLS), we tested whether grip strength is an independent predictor of frailty in a representative national population and confirmed their relationship.

The purpose of the study is to explore the relationship between grip strength and frailty in middle-aged and older Chinese adults using the CHARLS dataset and to select the optimal interpretable ML model to predict frailty in middle-aged and older adults. The SHapley Additive exPlanation (SHAP) tool was used to explain the optimal model for predicting frailty in middle-aged and older adults, to explore independent risk factors for frailty, and to provide individualized strategies for the management and prevention of frailty in middle-aged and older adults.

## 2 Materials and methods

### 2.1 Study design and population

Our study data were extracted from the CHARLS wave 2 (2013) dataset, which is openly available at http://charls.pku.edu.cn/. CHARLS is a nationally representative longitudinal research cohort in China that collects high-quality data on households and individuals of middle-aged and older adults aged 45 years and above to analyze population aging. As described by Liu Y et al. CHARLS began in 2011–2012 with a baseline survey in 28 provinces, 150 counties, 450 villages, and 10,257 households across the country. Respondents were assessed every 2 years with face-to-face personal interviews, sociodemographics, lifestyle, anthropometric measurements, and laboratory analysis (14).

**Figure 1:**
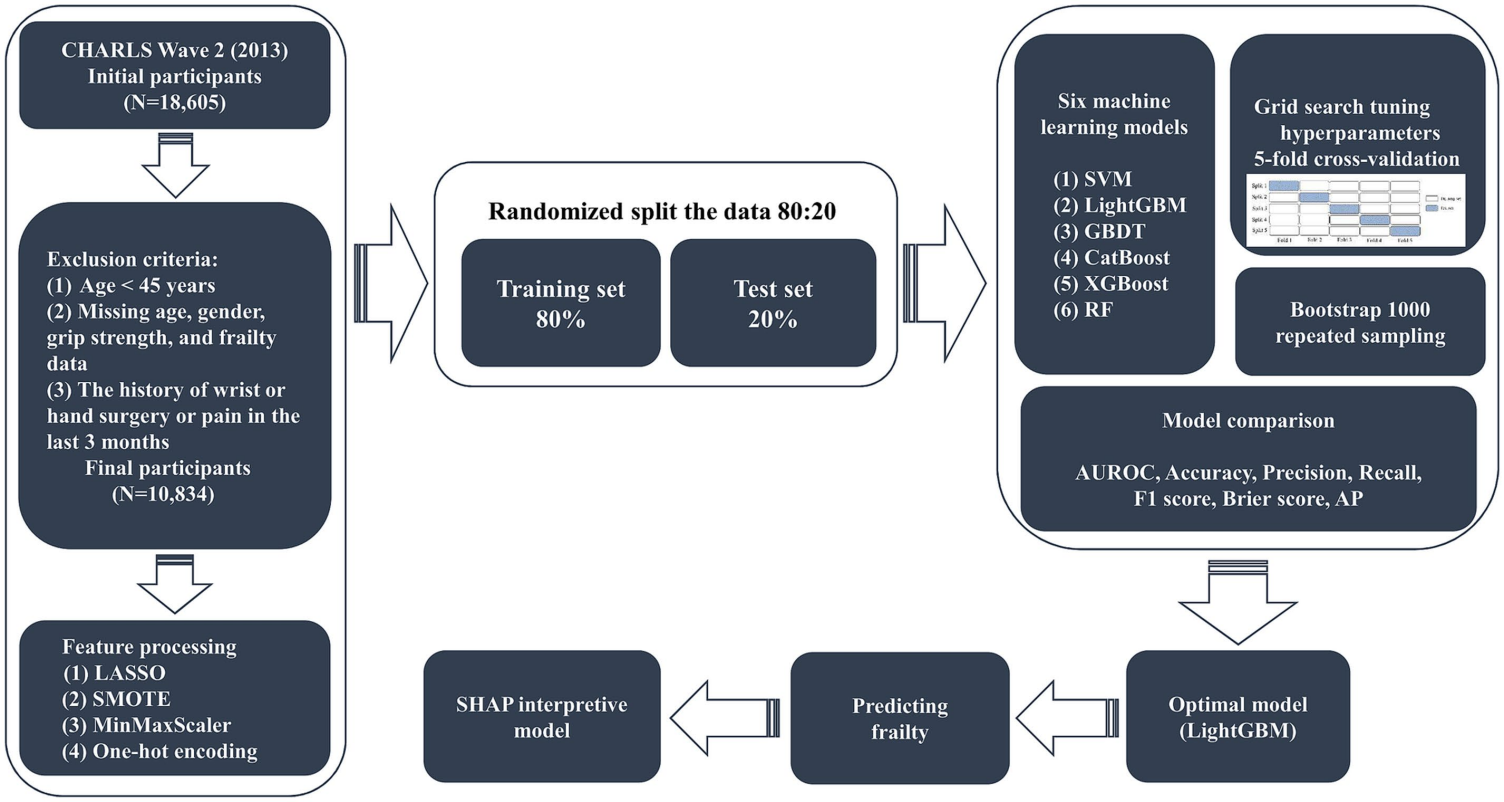
Study participants’ selection and design flowchart.

Since the CHARLS study in wave 2 (2013) has more complete information on the variables, we chose this survey cycle as the data source for our study. A total of 18,605 people were collected in the initial survey, and 10,834 participants were eventually included in the study after gradual screening. The inclusion criteria were as follows: (1) the age ≥ 45 years; (2) completed grip strength tests in both hands; and (3) obtained informed consent. Exclusion criteria were (1) the age < 45 years; (2) missing age, gender, grip strength, and frailty data; and (3) the history of wrist or hand surgery or pain in the last 3 months. The screening process for study participants and the study design are shown in Figure 1.

### 2.2 Ethics statement

The study procedures followed the Declaration of Helsinki, and the CHARLS study was approved by the Ethics Committee of Peking University (Approval No. IRB00001052-11015), and all participants signed the written informed consent. We followed the CHARLS guidelines for data use for study analysis only. All participants’ protected personal information is anonymized during our use of CHARLS data.

### 2.3 Feature extraction

**Figure 2:**
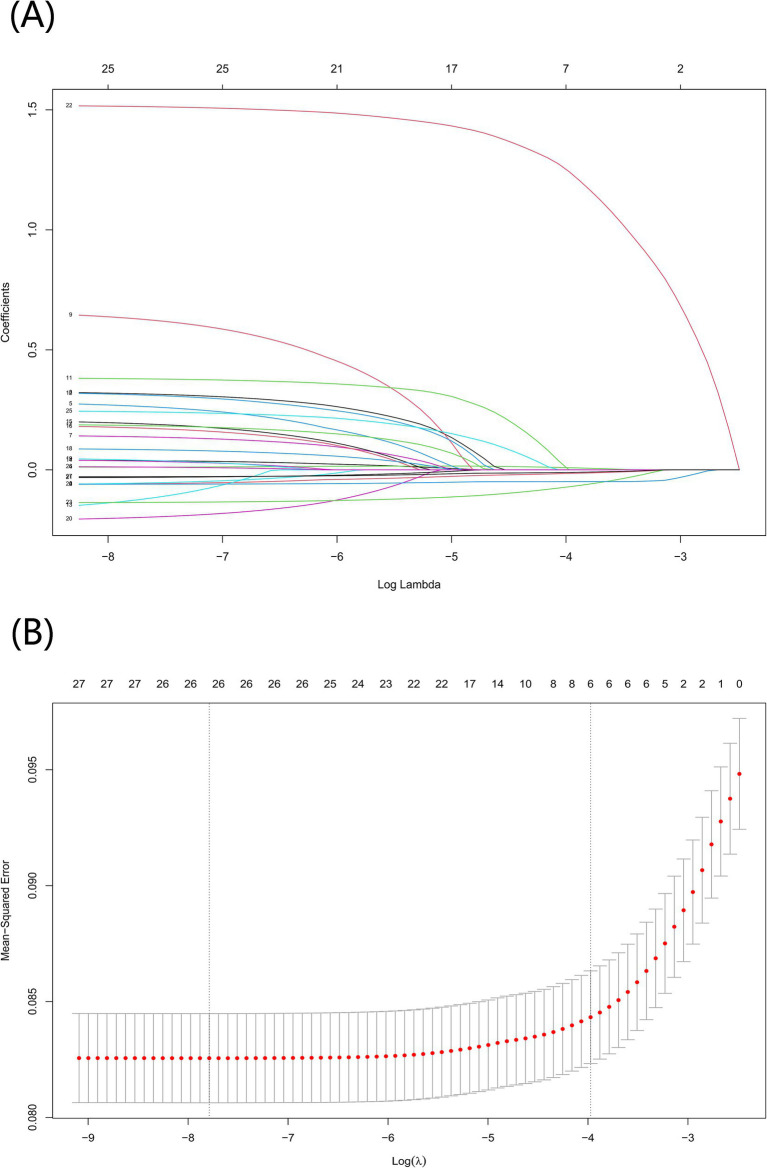
Screening for optimal model variables using the LASSO regression model. **(A)** To generate coefficient curves based on log(*λ*) sequences and to create nonzero coefficients by optimal λ. **(B)** The best λ in the LASSO model was selected by 10-fold cross-validation and using a minimum value criterion. The binomial deviation curves relative to the log(λ) are plotted and a virtual vertical line is drawn at the optimal value using one SE of the minimum criterion.

Based on previous studies (15–17) and expert opinions (three independent geriatricians from the Second Affiliated Hospital of Wenzhou Medical University), we selected 31 variables including demographics, anthropometric measurements, lifestyles, medical histories, laboratory analyses, socioeconomic information, and psychiatric interview data (Supplementary material 1) as the original candidates for the prediction of frailty model. We excluded candidate variables with more than 15% missing values, including tobacco use and activities of daily living (ADL) score. To avoid overfitting and multicollinearity, we used the least absolute shrinkage and selection operator (LASSO) regression to screen candidate predictor variables for predicting the frailty model (18, 19). We used 10-fold cross-validation to confirm the appropriate tuning parameter (*λ*) of the LASSO regression analysis to screen the optimal predictor frailty model variables and the predictor variables with a *p*-value less than 0.05 were used as the final predictor frailty model variables (Figure 2). For this reason, we removed marital status, hypertension, diabetes, cancer, heart disease, stroke, dyslipidemia, liver disease, kidney disease, asthma, alcohol consumption, tobacco use, insurance, social activities, depression, ADL score, orientation, and pain. After variable screening, inclusion in the final predictive frailty model consisted of 13 features and 1 label, with 5 categorical and 8 continuous variables.

### 2.4 Definition of features and the label

Diagnostic components of frailty based on the description of Bu F et al. include fatigue, weakness, decreased physical activity, weight loss, and slow movement, and frailty is defined as meeting 3 or more of the 5 indicators (20). Whereas those who meet 1 or 2 indicators are pre-frail, those who meet 0 are non-frail (21). Nighttime sleep duration was obtained by asking the question “During the past month, how many hours of actual sleep did you get at night (average hours for one night)?.” Poor sleep quality was assessed based on “my sleep was restless” and was categorized into 4 groups. Chronic diseases (hypertension, diabetes, cancer, chronic lung disease, heart disease, stroke, mental disease, arthritis or rheumatism, dyslipidemia, liver disease, kidney disease, digestive disease, and asthma) and pain were defined by self-report. The Katz Activities of Daily Living (ADL) score was used to assess the ability to perform activities of daily living, which are included in the CHARLS questionnaire: eating, dressing, transferring, toileting, bathing, and continence (22). Cognitive functioning was assessed through visuospatial skills, memory, orientation, and attention (23). The specific methods were as follows: (1) visuospatial skills were assessed by redrawing two overlapping pentagons; (2) memory was measured by the average score of immediate and delayed recall of 10 Chinese words; (3) orientation and attention were measured by cognitive status telephone interview, which was calculated based on responses to questions about the year, month, day, week, and season. The sum of the above items is the total cognitive functioning score, and the score ranges from 0 to 21, the higher the score the better the cognitive functioning (24). Mental health status was assessed using the Center for Epidemiologic Studies Depression Scale (CES-D) as described by Bergenfeld I et al. with a total score of 30 and depression defined as 10 and above (25).

### 2.5 Handling of missing values

We observed that the presence of missing data for some variables is common in the CHARLS dataset, and that direct deletion of information about individuals with missing data can lead to wasted medical information and participant selection bias. To optimally preserve participant information and biological characteristics of the original dataset, we performed an algorithm based on the K Nearest Neighbors (KNN) for missing value interpolation using the DMwR2 (version0.0.2) R package for variables with less than 15% missing data. To validate the effectiveness of the KNN interpolation method, we compared it with the average interpolation and multiple interpolation methods on the missing dataset using the Root Mean Square Error (RMSE) and Mean Absolute Error (MAE) as the evaluation metrics. The KNN interpolation method performs well in preserving data integrity and minimizing the prediction error.

### 2.6 Predictive modeling strategy

We randomly split the original dataset into 80:20 to better evaluate the performance and generalization ability of the frailty prediction model on unknown data, where 80% of the data is used as the model training set (*N* = 8,667) and 20% of the data is used as the model testing set (*N* = 2,167). We trained the predictive frailty model using the training set data, tested the predictive frailty model on the test set data, and tuned the hyperparameters using grid search to improve the generalization performance of the predictive frailty model (26). To better represent the generalization ability of the predictive frailty model, we used 5-fold cross-validation to evaluate each hyperparameter combination of the predictive frailty model as a way to identify and select the optimal hyperparameter combination (27). Since the prevalence of frailty in the participants of this study was only 10.61%, the data had class-imbalanced characteristics, which tends to bias the accuracy towards more classes when predicting frailty using ML models. To overcome such problems, we processed the class-imbalanced data for predicting frailty using the Synthetic Minority Oversampling Technique (SMOTE) to achieve class balance in the data (28). To eliminate the differences in the data magnitude of different features of the predictive frailty model and convert the data to a uniform scale, we normalized the continuous features using MinMaxScaler and converted the categorical features to a numerical form that can be used for the training of the predictive frailty model using one-hot coding (29, 30).

Different ML algorithms have different applicability properties and performance to the original dataset, so we used six ML models including SVM, Gradient Boosting Decision Tree (GBDT), LightGBM, Category Boosting (CatBoost), XGBoost, and RF to predict the grip strength-related frailty in middle age and older adults. The optimal prediction frailty model is identified based on the area under the receiver operating characteristic curve (AUROC) metrics of different prediction frailty models, and the optimal prediction frailty model is interpreted using the SHAP tool.

### 2.7 Interpretability tool for SHAP-based predictive frailty modeling

We used SHAP as an interpretable tool for optimal prediction of frailty models in middle-aged and older adults. Traditional variable importance only ranks the importance of the variables in predicting the frailty model and cannot explain how the variables affect the final frailty prediction of the model (31). SHAP is based on cooperative game theory, where the magnitude of the SHAP value accurately assesses the value of each feature’s contribution to the frailty prediction, and the positivity or negativity of the SHAP value suggests the directionality of the contribution (32).

### 2.8 Statistical analysis

Depending on whether the data conformed to a normal distribution, continuous variables were expressed as mean (standard deviation) or median (quartiles 1–3), and categorical variables were expressed as frequency (percentage). We divided all participants into two groups according to whether they were frail or non-frail. We compared each continuous variable between the two groups of patients using the Mann–Whitney U test, and compared differences in categorical variables using either the *χ*^2^ test or Fisher’s precision probability test. The relationship between grip strength and frailty was analyzed using multivariate logistic regression, trend tests, and interactions. We used Python (version 3.11.5) to analyze the ML models and evaluated the performance of each ML model using metrics such as AUROC, Accuracy, Precision, Recall, F1 Score, Brier Score, and Area under the Precise Recall (P-R) curve (AP). Comparisons between groups and logistic regression analysis of variables were statistically analyzed using R (version 4.3.1). The R packages we used include haven, gtsummary, DMwR2, dplyr, plyr, interactions, tidyverse, caret, arsenal, glmnet, and ggplot2. We used the Python libraries Scikit-learn (version 1.2.2) and imblearn library (version 0.10.1). The difference was considered statistically significant with a two-sided *p* value <0.05.

## 3 Results

### 3.1 Baseline characteristics of the participants

Based on the inclusion and exclusion criteria, the final study cohort included a total of 10,834 study participants, of which females: 5,749, and males: 5,085 were included in the baseline frailty status analysis, and the baseline characteristics of these participants are shown in Table 1. The prevalence of frailty in the present study was 10.61% (1,150/10,834) and the patients with frailty were older, with 22.09% being ≥75 years of age. Frail patients were more often unmarried (22.70%) and more often in poor self-perceived health (59.00%). The incidence of frailty was 62.00% in females and 38.00% in males, which is a significant difference. In the frail group, height, weight, body mass index (BMI), alcohol consumption, social activities, nighttime sleep duration, ADL score, orientation, cognitive function, waistline, and grip strength were significantly lower than that of the non-frail group, and the difference was statistically significant (*p* < 0.01). Hypertension, diabetes, cancer, chronic lung disease, heart disease, stroke, mental disease, arthritis or rheumatism, liver disease, kidney disease, disability, insurance, poor sleep quality, depression, poor life satisfaction, poor hearing, poor vision, and pain were significantly higher in the frail group than in the non-frail group. Disease, digestive disease, asthma, insurance, poor sleep quality, depression, poor life satisfaction, poor hearing, poor vision, and pain were significantly higher than those of the non-frail group, and the difference was statistically significant (*p* < 0.01).

**Table 1:** Baseline characteristics of study participants.

Characteristic	Non-frail (*N* = 9,684)	Frail (*N* = 1,150)	*p*-value
Height (cm)	158 (152, 164)	155 (149, 161)	<0.001
Weight (kg)	59 (52, 67)	52 (45, 61)	<0.001
BMI (kg/m^2^)	23.7 (21.4, 26.2)	21.9 (18.5, 24.8)	<0.001
Age (years)	60 (53, 66)	64 (58, 73)	<0.001
Age group			<0.001
<55	2,812 (29.04%)	194 (16.87%)	
55–64	3,834 (39.59%)	406 (35.30%)	
65–74	2,205 (22.77%)	296 (25.74%)	
≥75	833 (8.60%)	254 (22.09%)	
Gender			<0.001
Female	5,036 (52.00%)	713 (62.00%)	
Male	4,648 (48.00%)	437 (38.00%)	
Marital status			<0.001
Unmarried	1,214 (12.54%)	261 (22.70%)	
Married	8,470 (87.46%)	889 (77.30%)	
Self-perceived health status			<0.001
Fair	5,017 (51.88%)	364 (32.36%)	
Good	2,335 (24.14%)	95 (8.44%)	
Poor	2,319 (23.98%)	666 (59.20%)	
Hypertension			<0.001
No	7,142 (73.75%)	777 (67.57%)	
Yes	2,542 (26.25%)	373 (32.43%)	
Diabetes			<0.001
No	8,985 (92.78%)	1,031 (89.65%)	
Yes	699 (7.22%)	119 (10.35%)	
Cancer			<0.001
No	9,616 (99.30%)	1,130 (98.26%)	
Yes	68 (0.70%)	20 (1.74%)	
Chronic lung disease			<0.001
No	8,817 (91.05%)	985 (85.65%)	
Yes	867 (8.95%)	165 (14.35%)	
Heart disease			<0.001
No	8,599 (88.80%)	926 (80.52%)	
Yes	1,085 (11.20%)	224 (19.48%)	
Stroke			<0.001
No	9,497 (98.07%)	1,103 (95.91%)	
Yes	187 (1.93%)	47 (4.09%)	
Mental disease			0.010
No	9,594 (99.07%)	1,130 (98.26%)	
Yes	90 (0.93%)	20 (1.74%)	
Arthritis or rheumatism			<0.001
No	6,831 (70.54%)	687 (59.74%)	
Yes	2,853 (29.46%)	463 (40.26%)	
Dyslipidemia			0.400
No	8,698 (89.82%)	1,023 (88.96%)	
Yes	986 (10.18%)	127 (11.04%)	
Liver disease			0.010
No	9,370 (96.76%)	1,096 (95.30%)	
Yes	314 (3.24%)	54 (4.70%)	
Kidney disease			<0.001
No	9,159 (94.58%)	1,048 (91.13%)	
Yes	525 (5.42%)	102 (8.87%)	
Digestive disease			<0.001
No	7,652 (79.02%)	811 (70.52%)	
Yes	2,032 (20.98%)	339 (29.48%)	
Asthma			<0.001
No	9,430 (97.38%)	1,096 (95.30%)	
Yes	254 (2.62%)	54 (4.70%)	
Alcohol consumption			<0.001
No	6,319 (65.30%)	871 (77.49%)	
Yes	3,358 (34.70%)	253 (22.51%)	
Tobacco use			0.500
No	5,522 (93.70%)	665 (93.01%)	
Yes	371 (6.30%)	50 (6.99%)	
Insurance			0.002
No	9,373 (96.79%)	1,093 (95.04%)	
Yes	311 (3.21%)	57 (4.96%)	
Social activities			<0.001
No	3,853 (39.79%)	564 (49.04%)	
Yes	5,831 (60.21%)	586 (50.96%)	
Poor sleep quality			<0.001
Rarely or none of the time	4,922 (52.28%)	287 (29.41%)	
Some or a little of the time	1,534 (16.29%)	144 (14.75%)	
Occasionally or a moderate amount of the time	1,298 (13.79%)	198 (20.29%)	
Most or all of the time	1,661 (17.64%)	347 (35.55%)	
Nighttime sleep duration (h)	6.00 (5.00, 7.50)	5.50 (4.00, 7.00)	<0.001
Depression			<0.001
No	6,419 (72.26%)	208 (25.09%)	
Yes	2,464 (27.74%)	621 (74.91%)	
ADL score	6 (6, 6)	6 (6, 6)	<0.001
Orientation	4 (3, 5)	3 (1, 4)	<0.001
Cognitive function	11.5 (8.0, 14.0)	8.0 (4.5, 11.5)	<0.001
Life satisfaction			<0.001
Fair	6,821 (70.44%)	719 (62.52%)	
Good	2,386 (24.64%)	165 (14.35%)	
Poor	477 (4.93%)	266 (23.13%)	
Hearing			<0.001
Fair	4,488 (47.27%)	461 (45.24%)	
Good	3,747 (39.47%)	266 (26.10%)	
Poor	1,259 (13.26%)	292 (28.66%)	
Vision			<0.001
Fair	2,965 (31.22%)	345 (33.82%)	
Good	4,540 (47.81%)	332 (32.55%)	
Poor	1,991 (20.97%)	343 (33.63%)	
Pain			<0.001
Yes	3,209 (33.14%)	608 (52.87%)	
No	6,475 (66.86%)	542 (47.13%)	
Waistline (cm)	87 (80, 94)	84 (76, 91)	<0.001
Grip strength (kg)	29 (22, 36)	22 (16, 30)	<0.001

### 3.2 Multivariate logistic regression analysis of grip strength and the risk of frailty

To ensure that a wide range of variations in different grip strength levels could be captured while avoiding bias due to the subjective setting of thresholds, we explored thresholds and dose–response relationships for grip strength and risk of frailty by stratifying grip strength by quartiles (33). Table 2 presents the results of a multivariate logistic regression analysis examining the association between grip strength and the risk of frailty in males. In Model I, a negative association was observed between grip strength and frailty risk, with higher grip strength associated with a reduced risk (OR = 0.932, 95% CI: 0.923–0.942, *p* < 0.001). This association remained statistically significant in Model II after adjusting for age (OR = 0.939, 95% CI: 0.929–0.950, *p* < 0.001). In Model III, which included full adjustment for covariates, the association persisted, with each unit increase in grip strength corresponding to a 5.7% decrease in frailty risk (OR = 0.943, 95% CI: 0.931–0.954, *p* < 0.001). Moreover, in the fully adjusted Model III, males in the highest quartile of grip strength demonstrated a 63.9% lower risk of frailty compared to those in the lowest quartile (OR = 0.361, 95% CI: 0.252–0.509, *p* < 0.001). In addition, to explore whether grip strength interacted with other important variables, we analyzed the interaction of grip strength with the top three important variables of the SHAP summary plot of the optimal model, respectively. The results showed that there was an interaction effect between grip strength and cognitive function (*p* < 0.01). Table 3 presents the analysis of the association between female grip strength and the risk of frailty. In Model I, a negative association was identified, with higher grip strength associated with a reduced risk of frailty (OR = 0.928, 95% CI: 0.917–0.939, *p* < 0.001). This association remained statistically significant in Model II after adjusting for age (OR = 0.947, 95% CI: 0.935–0.958, *p* < 0.001). In Model III, after full adjustment for covariates, a consistent 4.9% reduction in frailty risk was observed for each unit increase in grip strength (OR = 0.951, 95% CI: 0.939–0.963, *p* < 0.001). Furthermore, in the fully adjusted Model III, individuals in the highest quartile of grip strength exhibited a 54.3% lower risk of frailty compared to those in the lowest quartile (OR = 0.457, 95% CI: 0.351–0.590, *p* < 0.001).

**Table 2:** Multivariate logistic regression analysis of grip strength and the risk of frailty in males.

Exposure	Model I		Model II		Model III	
	OR (95%CI)	*p*-value	OR (95%CI)	*p*-value	OR (95%CI)	*p*-value
Grip strength (continuous)	0.932 (0.923, 0.942)	<0.001	0.939 (0.929, 0.950)	<0.001	0.943 (0.931, 0.954)	<0.001
Grip strength (quartiles)						
Q1 (≤29.00)	Reference		Reference		Reference	
Q2 (>29.00, ≤35.25)	0.446 (0.345, 0.572)	<0.001	0.497 (0.382, 0.642)	<0.001	0.518 (0.398, 0.671)	<0.001
Q3 (>35.25, ≤41.25)	0.367 (0.279, 0.479)	<0.001	0.453 (0.338, 0.602)	<0.001	0.494 (0.368, 0.657)	<0.001
Q4 (>41.25, ≤67.63)	0.225 (0.162, 0.307)	<0.001	0.305 (0.214, 0.429)	<0.001	0.361 (0.252, 0.509)	<0.001
*p* for trend	<0.001		<0.001		<0.001	
Grip strength	Cognitive function		Nighttime sleep duration		BMI	
*p* for interaction	<0.001		0.231		0.980	

OR, odds ratio; CI, confidence intervals; Model I: no covariate adjustment; Model II: Adjusted for age; Model III: adjusted for age, chronic lung disease, mental disease, arthritis or rheumatism, digestive disease, nighttime sleep duration, and waistline.

**Table 3:** Multivariate logistic regression analysis of grip strength and the risk of frailty in females.

Exposure	Model I		Model II		Model III	
	OR (95%CI)	*p*-value	OR (95%CI)	*p*-value	OR (95%CI)	*p*-value
Grip strength (continuous)	0.928 (0.917, 0.939)	<0.001	0.947 (0.935, 0.958)	<0.001	0.951 (0.939, 0.963)	<0.001
Grip strength (quartiles)						
Q1 (≤19.00)	Reference		Reference		Reference	
Q2 (>19.00, ≤23.50)	0.461 (0.376, 0.564)	<0.001	0.556 (0.450, 0.685)	<0.001	0.565 (0.456, 0.698)	<0.001
Q3 (>23.50, ≤28.25)	0.306 (0.243, 0.382)	<0.001	0.409 (0.321, 0.519)	<0.001	0.426 (0.334, 0.542)	<0.001
Q4 (>28.25, ≤61.38)	0.279 (0.220, 0.352)	<0.001	0.405 (0.313, 0.521)	<0.001	0.457 (0.351, 0.590)	<0.001
*p* for trend	<0.001		<0.001		<0.001	
Grip strength	Cognitive function		Nighttime sleep duration		BMI	
*p* for interaction	<0.001		<0.010		0.825	

OR, odds ratio; CI, confidence intervals; Model I: no covariate adjustment; Model II: Adjusted for age; Model III: adjusted for age, chronic lung disease, mental disease, arthritis or rheumatism, digestive disease, nighttime sleep duration, and waistline.

### 3.3 Performance evaluation and comparison of six ML models

**Figure 3:**
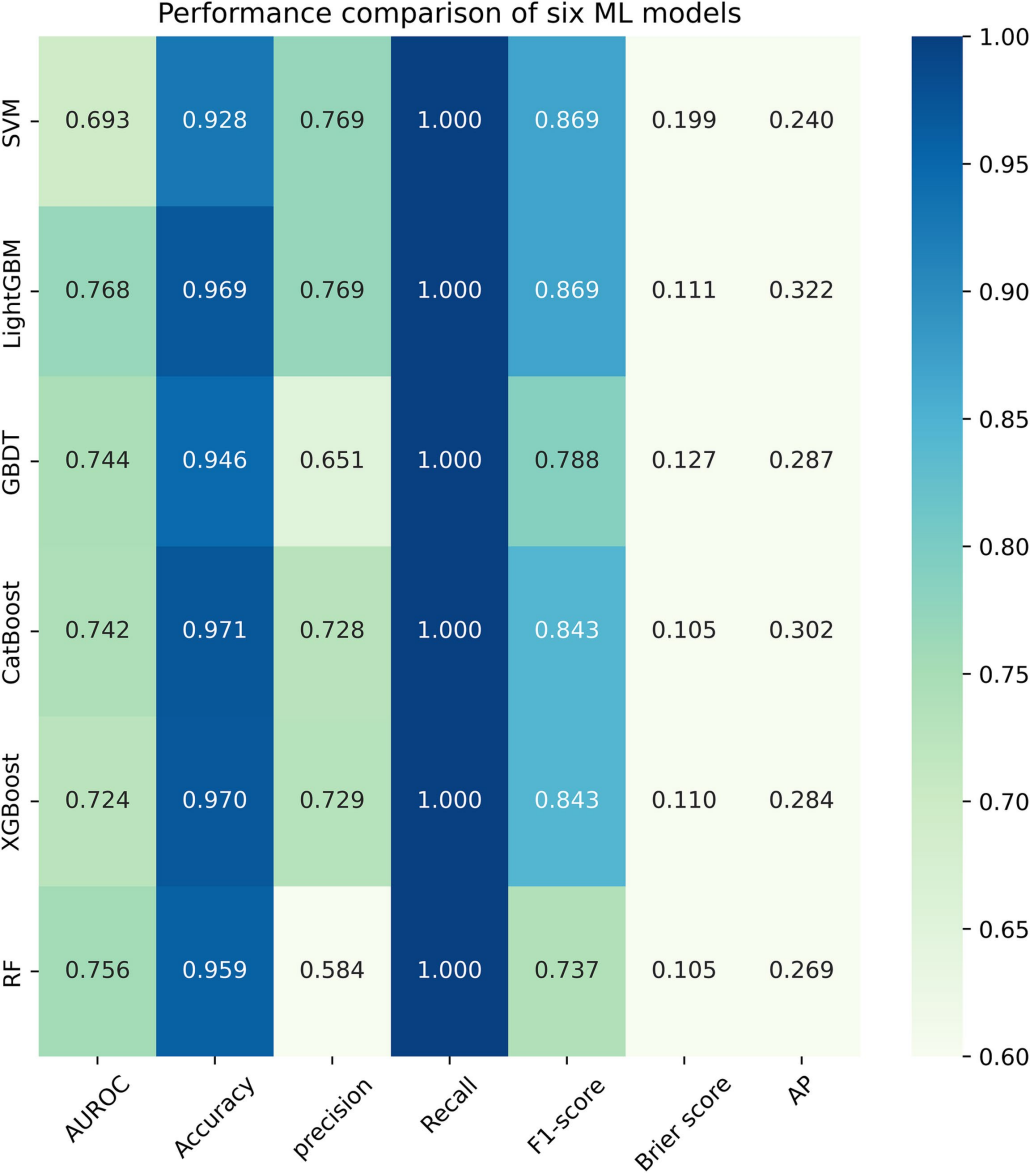
Heatmap comparing the performance of six ML models.

**Figure 4:**
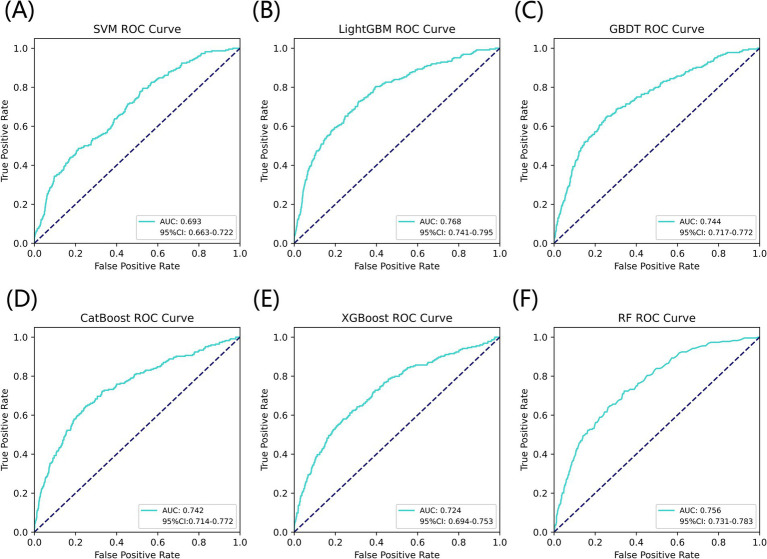
AUROC comparison of six ML models for predicting frailty. **(A)** SVM, **(B)** LightGBM, **(C)** GBDT, **(D)** CatBoost, **(E)** XGBoost, and **(F)** RF.

**Figure 5:**
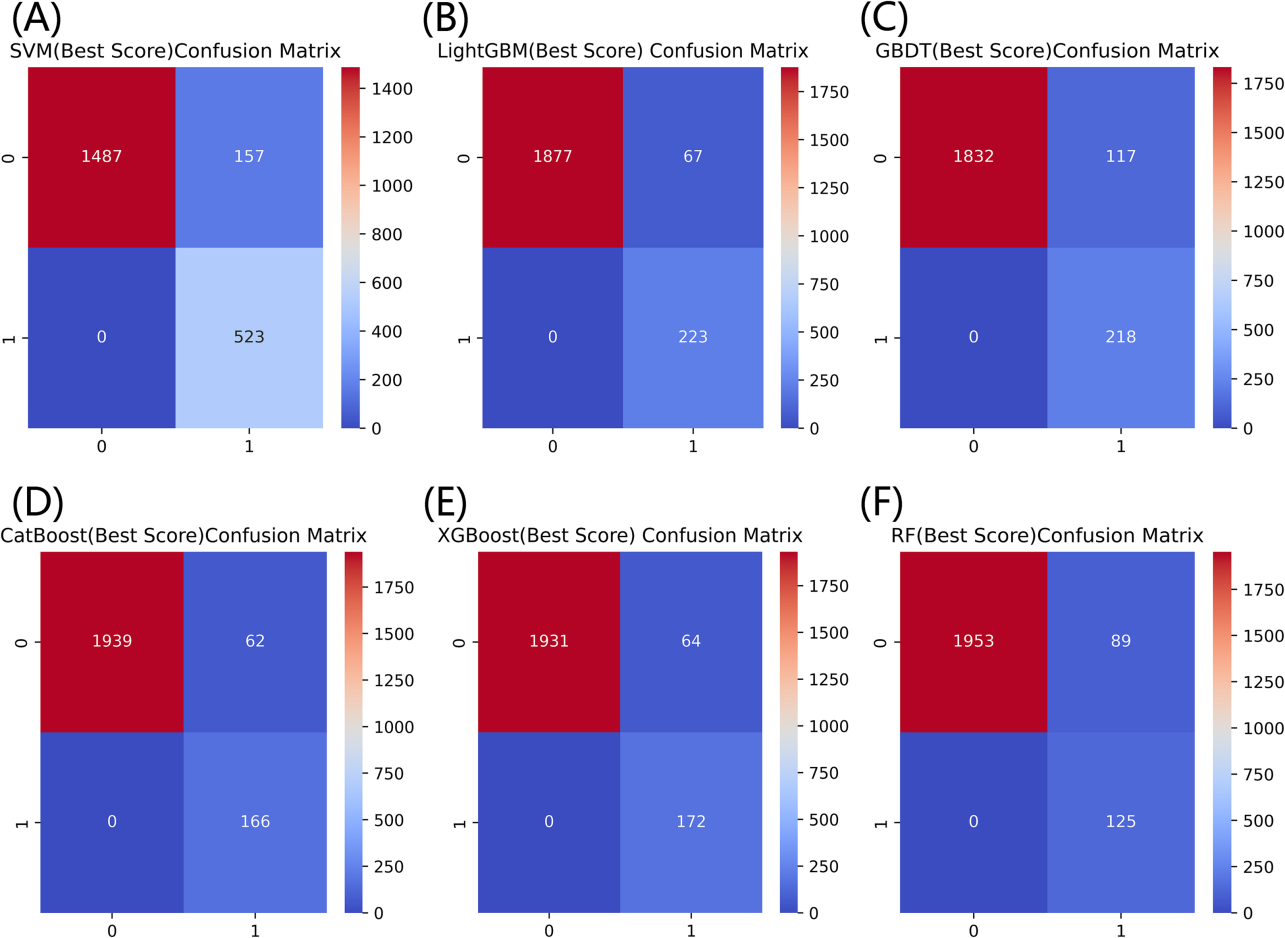
Comparison of confusion matrices for six models for predicting frailty. **(A)** SVM, **(B)** LightGBM, **(C)** GBDT, **(D)** CatBoost, **(E)** XGBoost, and **(F)** RF.

**Figure 6:**
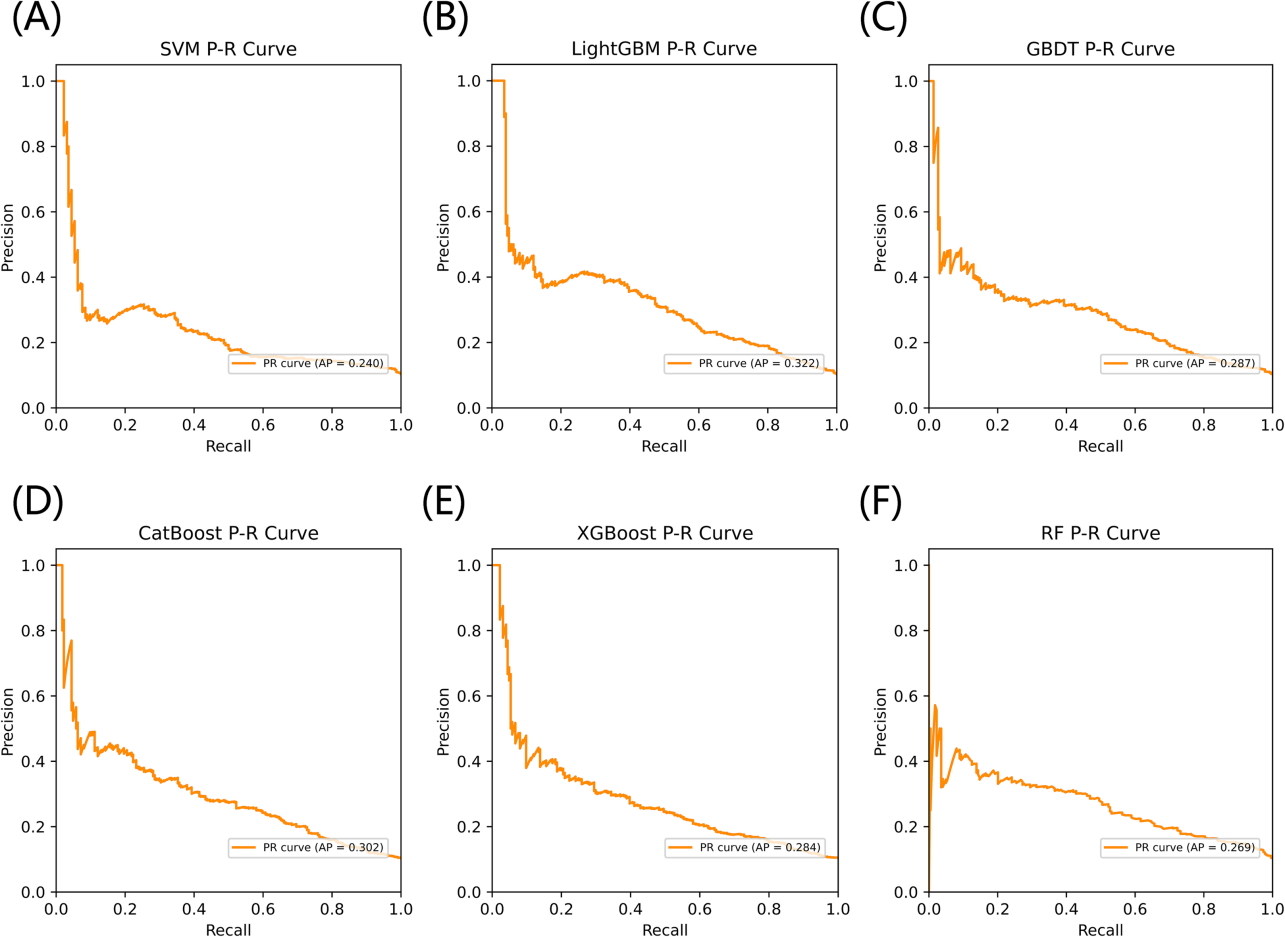
The AP comparison of six models for predicting frailty. **(A)** SVM, **(B)** LightGBM, **(C)** GBDT, **(D)** CatBoost, **(E)** XGBoost, and **(F)** RF.

Figure 3 listed seven performance discriminators for SVM, LightGBM, GBDT, CatBoost, XGBoost, and RF models used to predict frailty. We trained six predictive frailty ML models on the training set data and tested each predictive frailty model on the test set data for seven performance discriminators, including AUROC, accuracy, precision, recall, F1 score, Brier score, and AP. In addition, we performed 1,000 resamplings using Bootstrap to calculate the AUROC 95% CI for each predicted frailty model. Based on AUROC, the LightGBM model had the best predictive frailty performance compared to the other five models with an AUROC of 0.768 and 95% CI of 0.741–0.795 (Figure 4). According to the confusion matrix, the LightGBM model predicts frailty with high accuracy and robustness with accuracy, precision, recall, F1 score, Brier score, and AP of 96.9, 76.9, 100%, 0.869, 0.111, and 0.322, respectively, (Figure 5; Figure 6). Ultimately, we use the LightGBM model as the optimal model for predicting frailty and further interpret the model using the SHAP.

### 3.4 Interpreting the LightGBM model for predicting frailty using SHAP summary plot

**Figure 7:**
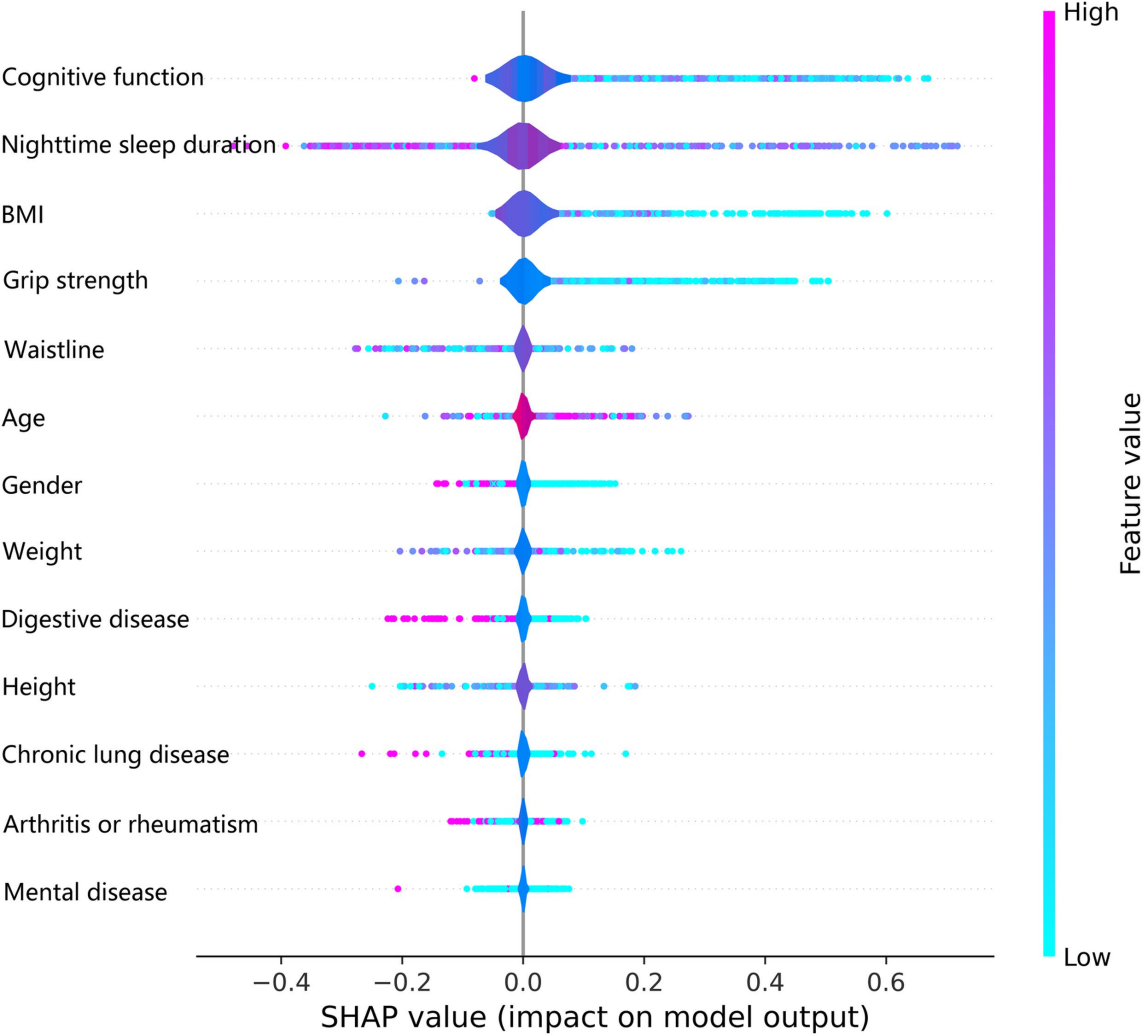
The SHAP global summary plot for the LightGBM model.

The SHAP summary plot listed the SHAP values of each feature, which was used to obtain the magnitude of each feature’s contribution to the LightGBM model’s prediction of frailty, and ordered the importance of each feature from highest to lowest (Figure 7). Cognitive function was the most important feature of the LightGBM model for predicting frailty, followed by nighttime sleep duration and BMI. Higher feature values in the SHAP summary plot are indicated by a reddish color, and a bluish color indicates lower feature values and the horizontal direction represents whether the SHAP value of the feature has a positive or negative effect on the prediction of frailty by the LightGBM model. We observed from the SHAP summary plots that a decreased value of the cognitive function feature has a positive effect on the model predicting frailty, while an increased value of the nighttime sleep duration feature has a negative effect on the model predicting frailty.

### 3.5 The SHAP individual force plot for the predicted frailty LightGBM model

**Figure 8:**
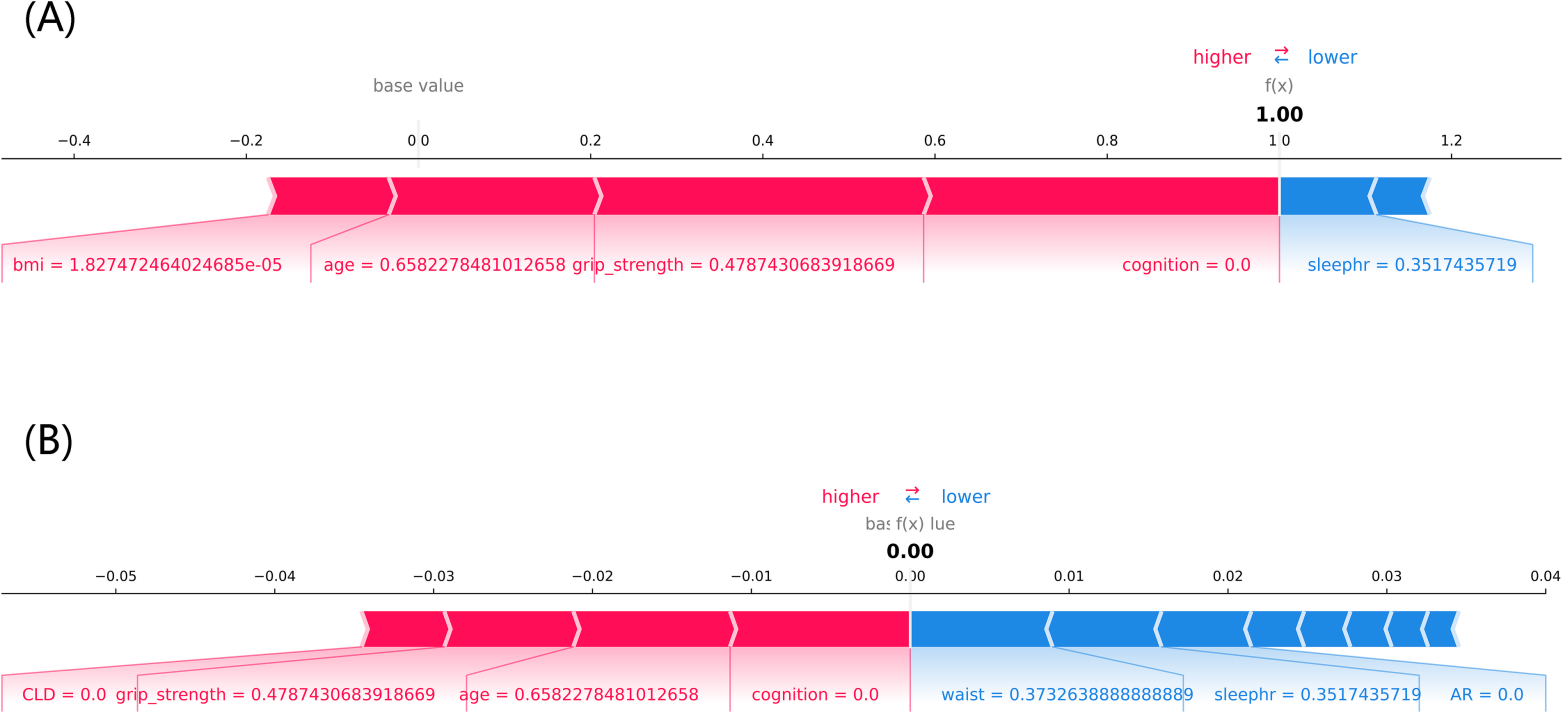
The SHAP force plot of individuals with different frailty prediction outcomes. **(A)** frailty, **(B)** non-frailty, cognition: cognitive function, sleephr: nighttime sleep duration, CLD: chronic lung disease, waist: waistline.

Figure 8 shows the individual force plots for (A) non-frailty and (B) frailty. The SHAP value for each feature is labeled below the arrows, indicating the contribution of each feature to the model’s frailty prediction, and the number on the horizontal axis is the probabilistic prediction value f(x). The red arrows on the left side represent features that positively influence the model’s frailty prediction, the blue arrows on the right side represent features that negatively influence the model’s frailty prediction, and the length of the arrows indicates the magnitude of the feature’s contribution value to the model’s frailty prediction. Figure 8A demonstrates that the LightGBM model predicted a frail individual, and Figure 8B indicates that the LightGBM model predicted a non-frail individual.

## 4 Discussion

This study is the first to explore the relationship between grip strength and frailty in middle-aged and older adults using the interpretable ML method, which showed a prevalence of frailty of 10.61%, which is consistent with the previously reported prevalence of frailty of 4.6% ~ 17.1% (34). Studies have shown that frailty is closely associated with hospitalizations, falls, fractures, and death, so accurately identifying those at risk for frailty is important for preventing frailty and related adverse outcomes (35). Our analysis revealed a negative association between grip strength and the risk of frailty, with this relationship becoming evident when grip strength exceeded 29.00 kg in males and 19.00 kg in females. This finding suggests the presence of a grip strength threshold for assessing frailty, which varies by gender. Specifically, grip strength is negatively correlated with frailty risk and can serve as an indicator of frailty only when it exceeds 29.00 kg in males and 19.00 kg in females. We constructed the SHAP-based interpretable lightGBM model to predict grip strength-related frailty in the middle-aged and older adults. In addition, we trained five other ML models, among which the LightGBM model has the optimal performance. The performance metrics of the LightGBM model, including AUROC, accuracy, precision, recall, F1 score, Brier score, and AP, were 0.768 (95% CI, 0.741–0.795), 96.9, 76.9, 100%, 0.869, 0.111, and 0.322, respectively. These results demonstrate the model’s high reliability, robustness, and accuracy in predicting frailty. The model demonstrated moderate to good discriminatory ability in predicting frailty, as an AUROC between 0.7 and 0.8 is generally regarded as acceptable in medical prediction tasks (36).

The LightGBM model’s superior performance is rooted in its efficient computational design and advanced feature handling capabilities. By using histogram-based learning, LightGBM significantly reduces computational overhead, making it faster and more memory-efficient than other gradient-boosting frameworks like XGBoost. Its leaf-wise tree growth strategy enhances the model’s ability to identify complex interactions between variables, leading to higher predictive accuracy. Moreover, the integrated regularization mechanisms effectively prevent overfitting, a critical advantage when working with imbalanced datasets like ours. These factors collectively contribute to the model’s robustness and reliability, underscoring its suitability for predicting frailty in middle-aged and older populations. Using SHAP individual force plots to interpret the LightGBM model allows for a better understanding of the decision-making process for model prediction frailty. Using the SHAP global summary plot, we also identified and ranked the importance of significant risk factors that predict frailty in the middle-aged and older adults. Cognitive function is the most important predictor variable for the LightGBM model, followed by nighttime sleep duration, BMI, and grip strength. Ma W et al. also confirmed the existence of a significant association between frailty and cognitive function and found that depression partially mediated the link between frailty and cognitive function (37).

Using the interpretable LightGBM model to predict frailty, we found that lower grip strength, cognitive function, BMI, and nighttime sleep duration were associated with a higher risk of frailty in middle-aged and older adults. Older age and female gender were also associated with a higher risk of frailty. A cross-sectional study of the relationship between frailty and muscle response by Suzuki Y et al. showed that grip reaction speed decreases with frailty, and that measuring grip strength in both hands can help to study the relationship between frailty and grip strength (38). A review by Vaishya R et al. stated that hand grip strength is a basic indicator for assessing muscle function and overall body function, is particularly relevant to old age, identifies a variety of health problems, and is an important biomarker of health (39). A cross-sectional observational study by Choe et al. found that muscle mass did not directly affect frailty and that grip strength mediated the relationship between muscle mass and frailty in older Korean adults (40). A systematic review and meta-analysis by Robinson TL et al. noted that frailty has a significant and negative impact on cognitive function, and their combined effect may increase the risk of adverse health outcomes in older adults (41). Jayanama K et al. examined the relationship between high BMI and frailty and mortality and found that being overweight or obese was associated with a higher risk of frailty and that the relationship between BMI and frailty was partly due to body fat (42). Arias-Fernández L et al. studied the relationship between sleep disorders and impaired physical functioning in older adults and suggested that self-reported poor sleep quality was associated with frailty and muscle weakness (43). These points confirmed our findings, and we hypothesized that grip strength, cognitive function, BMI, and nighttime sleep duration could serve as independent predictors of frailty.

Gordon EH et al. suggested that aging is a major risk factor for most chronic diseases and that frailty is associated with many factors over the life course, particularly smoking, chronic disease, obesity, and economic deprivation (44). Bellelli F et al. showed that frailty increases with age and is associated with shorter years of education and that there is an exponential interaction between education and age in influencing frailty (45). Park C et al. and Zeidan RS et al. found that frailty is a manifestation of age-related decline in physiologic reserve and increased vulnerability and that there is a significant gender difference in the prevalence of frailty, with frailty being more common and severe in females, but with a relatively lower risk of death in females (3, 46). A cross-sectional study of frailty and gender differences in Europe found that females in Europe were more frail and had more comorbidities than males (47). These points are consistent with our findings and also suggest that age and gender may serve as independent predictors of frailty.

The interpretable LightGBM predictive model developed in this study demonstrates significant clinical utility in the management of frailty. For middle-aged and older adults, grip strength can be enhanced through strength training and nutritional interventions as part of personalized frailty management strategies. Given the observed relationship between nocturnal sleep duration and frailty risk, measures such as behavioral therapies, reduction in the use of central nervous system depressants, and optimization of the sleep environment may help improve sleep quality. Cognitive function, identified as a critical predictor of frailty, can be targeted through interventions like cognitive training and social engagement activities. For patients with excessively low body weight and muscle mass, maintaining a healthy BMI through dietary modifications and moderate exercise can mitigate the risk of frailty. The SHAP force plot offers a valuable tool for visualizing the contribution of individual features to a patient’s frailty risk. For instance, patients with low grip strength but high BMI may benefit from prioritized interventions focused on strength training and weight management. Furthermore, the SHAP global feature importance map highlights key predictors, such as cognitive function and sleep duration, which can be incorporated into clinical screening protocols to guide early intervention efforts. By leveraging SHAP values, specific frailty management pathways can be designed to identify high-risk individuals. These patients can then be allocated to tailored intervention programs, such as grip strength training or cognitive function rehabilitation. The interpretability of the model provides clinicians with actionable decision support, particularly when managing a large cohort of patients with limited resources. Through feature prioritization, the model facilitates the efficient allocation of intervention resources, ensuring that high-impact strategies are applied where they are most needed.

This study has some strengths and limitations regarding the prediction of frailty. First, this study is based on the CHARLS data, which is nationally representative and high-quality data. It also uses the big data analytic power of ML to predict frailty in China’s middle-aged and older adults. Second, the SHAP-based interpretable LightGBM model makes it easy for users to understand how it predicts frailty in middle-aged and older adults. Third, since the CHARLS data was a prospective study, it was possible to infer the existence of a causal relationship between the variables and the outcome, making the LightGBM model highly reliable in predicting frailty. The potential underrepresentation of rural populations in the CHARLS dataset introduces a limitation to our study. Rural communities often experience unique sociodemographic and health-related challenges, including lower socioeconomic status, limited healthcare access, and higher prevalence of malnutrition, which may influence the relationship between grip strength and frailty. Future studies should aim to validate our findings using datasets with a more balanced representation of rural and urban populations, or by employing stratified sampling techniques to minimize bias.

## 5 Conclusion

The grip strength-related LightGBM prediction model, constructed and tested based on SHAP, has high accuracy, robustness, and reliability in predicting the risk of frailty in middle-aged and older Chinese adults. The interpretable LightGBM predictive model we created accurately explores risk factors for frailty in middle-aged and older adults and has important clinical applications. Reducing grip strength, cognitive function, nighttime sleep duration, and BMI increases the risk of frailty and may provide strategies for individualized management of frailty in middle-aged and older adults.

## Data Availability

The original contributions presented in the study are included in the article/Supplementary material, further inquiries can be directed to the corresponding author.
