# Supplementary material for: Predicting grip strength-related frailty in middle-aged and older Chinese adults using interpretable machine learning models: a prospective cohort study
Source: Front Public Health. 2024 Dec 17;12:1489848. doi: 10.3389/fpubh.2024.1489848 (PMC11685125; doi:10.3389/fpubh.2024.1489848)
Supplement: Supplementary file 1 [file Table_1.DOCX]

The 31 original candidate variables included Height, Weight, BMI, Age, Gender, Marital status, Hypertension, Diabetes, Cancer, Chronic lung disease, Heart disease, Stroke, Mental disease, Arthritis or rheumatism, Dyslipidemia, Liver disease, Kidney disease, Digestive disease, Asthma, Alcohol consumption, Tobacco use, Insurance, Social activities, Nighttime sleep duration, Depression, ADL score, Orientation, Cognitive function, Pain, Waistline, Grip strength.
